# Sterile Insect Technique (SIT) and Its Applications

**DOI:** 10.3390/insects12070638

**Published:** 2021-07-13

**Authors:** Kostas Bourtzis, Marc J. B. Vreysen

**Affiliations:** Insect Pest Control Laboratory, Joint FAO/IAEA Centre of Nuclear Techniques in Food and Agriculture, A-2444 Seibersdorf, Austria

**Keywords:** area wide pest management, insect pest control, vector control, fruit flies, tsetse flies, mosquitoes, moths, Hemiptera

## Abstract

**Simple Summary:**

Insects represent the most speciose and abundant group of animals on this planet. Most species are beneficial, however, a small number of them are major plant pests or vectors of major pathogens in livestock and humans. Insecticides have been used for decades as the main weapon for the population control of insect pests. However, concerns about human health and the environment as well as the increased prevalence of insecticide resistance have called for more environment-friendly and sustainable insect pest control approaches such as the sterile insect technique (SIT).

**Abstract:**

Although most insect species have a beneficial role in the ecosystems, some of them represent major plant pests and disease vectors for livestock and humans. During the last six–seven decades, the sterile insect technique (SIT) has been used as part of area-wide integrated pest management strategies to suppress, contain, locally eradicate or prevent the (re)invasion of insect pest populations and disease vectors worldwide. This Special Issue on “Sterile insect technique (SIT) and its applications”, which consists of 27 manuscripts (7 reviews and 20 original research articles), provides an update on the research and development efforts in this area. The manuscripts report on all the different components of the SIT package including mass-rearing, development of genetic sexing strains, irradiation, quality control as well as field trials.

## 1. Introduction

Insects epitomize the most evolutionary successful animal group on Earth. They have adapted almost everywhere on our planet to diverse ecological conditions. The majority of insect species play an important and beneficial role in the ecosystems. However, some of them represent a significant threat for agricultural production as well as for animal and human health. For decades, application of broad-spectrum insecticides has been the main tool for the management of insect pest populations and disease vectors. The continuous and irrational use of these chemicals has resulted in widespread insecticide resistance while their negative impact on human health, food chain and the environment has been documented beyond any doubt. There is an urgent need for environment-friendly, species-specific, and sustainable approaches, such as the sterile insect technique (SIT) for the population control of insect species and disease vectors [1].

The concept of the SIT was conceived by Edward F. Knipling, and it was first used for the population control of the New World screwworm, *Cochliomyia hominivorax* in the 1950s [2]. Since then, it has been successfully used as a component of area-wide integrated pest management (AW-IPM) approaches [3], to suppress, contain, prevent the (re)introduction and even locally eradicate populations of insect pests and disease vectors [1,4]. This Special Issue on “Sterile insect technique (SIT) and its applications” presents recent progress in this area as well as the challenges SIT faces from the developmental phase until its implementation in the field. The Special Issue consists of 27 manuscripts (7 reviews and 20 original research articles) covering a wide range of components of the SIT package including mass-rearing, development of genetic sexing strains, irradiation, quality control as well as small-scale field trials and large-scale, operational population suppression programs (https://www.mdpi.com/journal/insects/special_issues/mr_sit accessed on 8 July 2021). The manuscripts present results and achievements from work focusing on all main groups of SIT target species such as plant pests (fruit flies, lepidoptera), livestock pests (tsetse flies and sheep blow fly), and human disease vectors (mosquitoes), and some unusual groups such as Hemiptera.

## 2. Fruit Flies

The development of robust and cost-effective (mass-)rearing systems is a prerequisite for the development and implementation of the SIT. Sassù and colleagues (2019) report on the development and comparative evaluation of a wax panel and a netted oviposition system as egg collection systems for the spotted wing drosophila, *Drosophila suzukii* [5]. The results presented clearly show that the wax panel system is more practical, less laborious and produces significantly more and higher quality eggs than the netted oviposition system. Pascacio-Villafán and colleagues (2020) used different ingredients as gelling agents in yeast-reduced gel diets indicating that only the ones with agar and carrageenan are cheaper and produce more and better-quality Mexican fruit flies *Anastrepha ludens* compared to the diet currently used in the Moscafrut mass-rearing facility [6]. In addition, the gel-carrageenan diet produces suitable larvae as hosts for the rearing of the parasitoid *Diachasmimorpha longicaudata*.

Pascacio-Villafán and colleagues (2021) also tested different pupation substrates and substrate volumes for the Mediterranean fruit fly *Ceratitis capitata* VIENNA 8 genetic sexing strain (GSS) that is currently used by almost all operational SIT programs against this major agricultural pest worldwide [7]. They concluded that cellulose III at low volumes is the best pupation substrate, with respect to productivity, quality and cost-effectiveness. Mastrangelo and colleagues (2021) report on an improved rearing protocol for the South American fruit fly *Anastrepha fraterculus* (Vacaria strain), including a novel larval diet in which agar has been replaced by carrageenan [8]. The new rearing protocol was efficient and cost-effective producing insects of high quality that can be used for SIT applications. Importantly, after two years of using this novel rearing protocol and larval diet, no evidence of sexual isolation has been observed between mass-produced insects and the wild target population.

Although SIT applications for fruit flies can be successfully implemented by releasing both sterile males and sterile females, it has been shown that the efficiency and cost-effectiveness can be increased significantly if only sterile males are released [9,10]. Ramirez and colleagues (2021) present a new GSS for *A. ludens*, namely GUA10 that is based on the *black pupae* (*bp*) gene [11]. Comparative analysis showed that the *A. ludens* GUA10 GSS exhibits better rearing efficiency, genetic stability and overall quality than the previously developed *A. ludens* TAP7 GSS. In addition, Nguyen and colleagues (2021) discuss how currently available knowledge about single point mutations of the vinegar fly *Drosophila melanogaster* genes, which are known to result to temperature sensitive phenotypes, can be exploited in order to induce similar mutations in the orthologous genes of insect pests and disease vectors for the construction of GSS in support of SIT and other insect pest control programs [12].

Several studies have shown that high quality sterile flies for SIT applications can be produced when the insects are irradiated under hypoxic (low oxygen) conditions. Giustina and colleagues (2020) reported that males of two *A. fraterculus* strains (a bisexual strain and a GSS) require a dose of 74 Gy administered under hypoxia to induce 99% sterility in females while an irradiation dose between 80–90 Gy can induce complete sterility [13]. On the other hand, females can be fully sterilized with an irradiation dose of 50 Gy or above.

The importance of proper quality control and evaluation of every step in an SIT program, particularly if it is at an operational level, is also highlighted in the manuscript by Pla and colleagues (2021) [14]. The authors review the significant achievements of the SIT population suppression program carried out against *C. capitata* by the Department of Agriculture and the TRAGSA company in Valencia, Spain. In 2007, the SIT was incorporated in an AW-IPM program to manage *C. capitata* populations with the main goal to drastically reduce insecticide use and to protect the production and export industry of citrus and other fresh fruits in the region. The results of the program are impressive: a >90% reduction in insecticide use and significant increase in the export of citrus and fresh fruits for the Valencia community.

## 3. Lepidoptera

Lepidoptera are, in addition to fruit flies, major agricultural pests and their populations can be very efficiently managed by AW-IPM programs that include an SIT component. Marec and Vreysen (2020) [15] discuss the characteristic cytogenetic features of Lepidoptera as well as their high resistance to ionizing radiation. The latter property has resulted in the development of the inherited sterility (IS) method, an SIT-derived technology, which is currently used to successfully suppress populations of several lepidopteran pest species. In addition, the authors report on the quality control analysis of (mass-) reared and released insects, which is the key for successful SIT/IS applications, and they also discuss how recent developments in genetics and genomics may facilitate the development of genetic sexing strains.

No SIT or any other insect pest control method can be applied and be successful if it is not socially acceptable. Paterson and colleagues (2020) [16] present a study which indicates the positive perception of a local community in Hastings, New Zealand in respect to hosting insect traps in their properties and to applying SIT against the codling moth *Cydia pomonella*. In an interesting field trial, Horner and colleagues (2020a) [17] report that an AW-IPM project in New Zealand, which was based on the initial reduction of target pest populations by mating disruption and insecticides followed by sterile male-only releases, suppressed codling moth populations with 67% to 99%. In addition, Horner and colleagues (2020b) [18] show that codling moth populations that remain at high population densities in unmanaged peri-urban sites pose a significant risk of immigration into treated orchards. They suggest that these populations need to be reduced by SIT applications in the frame of an AW-IPM suppression program. Esch and colleagues (2021) [19] showed that sterile codling moths can be successfully released by small uncrewed aircraft systems (UASs). The data presented clearly indicate that UAS-based releases achieved higher recapture rates as compared with ground releases and are much cheaper to operate than the commonly used fixed wing aircraft for the release of sterile moths.

Recently, a project has been initiated in Arica, Chile aiming at the development and implementation of the SIT, as part of an AW-IPM approach, to suppress populations of the European grapevine moth *Lobesia botrana*. Simmons and colleagues (2021) [20] review the activities of this program describing the advances (rearing, irradiation, packaging, transport, release and monitoring) as well as the challenges to achieve releases of high-quality sterile males at adequate overflooding ratios in non-isolated urban areas.

## 4. Hemiptera

Hemiptera are an unusual target insect group for the SIT, in view that all development stages and potential release stages can cause damage to crops. Despite this, efforts have been made to assess whether populations of some hemipteran pest species can be targeted by the SIT, and the brown marmorated stink bug *Halyomorpha halys* has been considered as a potential candidate by some researchers. Suckling and colleagues (2020a) [21] present a novel live (or lethal) trap for this insect pest species that is a cheaper and more efficient alternative to the currently available ones. These traps could be useful for AW-IPM approaches with an SIT component. In a separate study, Suckling and colleagues (2020b) [22] report that an irradiation dose of 16 Gy did not affect the mating competitiveness and competency of *H. halys* males and could be considered a suitable dose in SIT-based population suppression programs. The authors also note that dosimetry is important and follow-up studies need to be performed in order to explain the different sterility levels obtained by the same irradiation dose in different facilities. The authors further discuss the potential of collecting overwintering males from the field using the aggregation pheromone, and then sterilized and released them as an alternative to the more conventional pathway which is based on the (mass-)rearing in a facility, irradiation and then release of sterile insects. However, this “alternative” procedure may be challenging both at the application and regulatory level. Interestingly, and similar to the abovementioned study on *H. halys*, Horrocks and colleagues (2020) [23] show that the application of an irradiation dose of 16 Gy or higher could sterilize male green vegetable bug *Nezara viridula*, another hemipteran species, by >99%, while females required a dose of 28 Gy to become fully sterile.

## 5. Livestock Pests

The SIT has been successfully used for the suppression, and even the local eradication, of tsetse populations transmitting African trypanosomes causing sleeping sickness in humans and nagana in animals [4]. Mating performance of irradiated male insects is one of the key factors that need to be evaluated before the implementation of SIT releases. De Beer and colleagues (2020) [24] report that irradiation of *Glossina austeni* males from a 37 year-old colony as adults or late stage pupae to obtain ca. 99% sterility did not affect their mating performance, and it was concluded that these sterile males are suitable for SIT applications as shown in laboratory and semi-field experiments.

The SIT has also been proposed as a tool to control populations of another major livestock pest species, the Australian sheep blow fly *Lucilia cuprina*. Yan and colleagues (2020) [25] report on the development of a transgenic embryonic sexing system (TESS) that results in the elimination of females at the embryonic stage in the absence of tetracycline from the diet, which could potentially be used for male-only releases if regulatory approval is granted.

## 6. Mosquitoes

The SIT has been very successful to manage populations of plant and livestock pests worldwide [1,4]. More recently, the SIT has been considered as a tool to suppress mosquito species transmitting major human pathogens, such as the yellow fever mosquito *Aedes aegypti* and the Asian tiger mosquito *Ae. albopictus*, using a phased conditional approach [26,27]. Oliva and colleagues (2021) [28] present a roadmap and good practice framework for the design, implementation, and evaluation of pilot field trials aiming at the suppression of *Ae. aegypti* and *Ae. albopictus* populations using the SIT. This pathway is addressed not only to scientists but also to non-specialists including stakeholders, implementers, and decision-makers. Gouagna and colleagues (2020) [29] report on the SIT feasibility program developed and implemented in La Réunion island against *Ae. albopictus* and review all activities, from the laboratory to the field, carried out during the last ten years including a public awareness campaign and regulatory validation. As public acceptance and support is an important parameter for the successful implementation of an SIT program, Stefopoulou and colleagues (2021) [30] used a KAP (knowledge, attitude, practices) survey and carried out a door-to-door campaign as a prerelease action to inform the Vravrona community, Attica, Greece about an upcoming SIT trial to suppress an *Ae. albopictus* population in that area. The residents were very positive and supportive of the SIT project, and this was further confirmed by the reduction of the target mosquito population before the initiation of the sterile male releases, most likely due to the removal of mosquito habitats from residents’ yards.

Availability of entomological baseline data, ideally over a period of two years prior to an intervention, is also one of the prerequisites for SIT applications. Marina and colleagues (2021) [31] monitored the population dynamics of *Ae. aegypti* and *Ae. albopictus* for two years (2016–2018) in two rural villages in Chiapas, Mexico. Data collected were carefully evaluated and taken into consideration in the design of a small-scale SIT field trial against *Ae. aegypti*. Using mosquitoes from the Chiapas region, Bond and colleagues (2021) [32] investigated the effects of irradiation on the mating competitiveness of *Ae. aegypti* and *Ae. albopictus* males under both laboratory and field-cage conditions. The results clearly indicate that the produced sterile male *Ae. aegypti* and *Ae. albopictus* maintained their competitiveness and when treated with an irradiation dose of 50 and 40 Gy, respectively, they induced up to 88% sterility under field-cage conditions.

During the last years there has been an increase in developing and testing the SIT in the field against *Ae. aegypti* and *Ae. albopictus* populations in different parts of the world. Tur and colleagues (2021) [33] report on a recent initiative of the Agriculture Department of the Valencian Region, Spain to develop and implement a small-scale SIT field trial against the invasive mosquito species, *Ae. albopictus*. The authors present the current operating procedures and quality control parameters of the established medium-scale rearing facility, which has produced over 15 million irradiated sterile males for release during the last two years (2018–2020) with a minimal average female contamination of 0.17%.

Gato and colleagues (2021) [34] present the impressive results of an SIT field trial against *Ae. aegypti* in two sub-urban areas of Havana, Cuba. During a period of 20 weeks, more than 1.2 million sterile males were released over an inhabited target area of about 50 ha. The sterile males were highly competitive, induced high levels of sterility and managed to fully suppress the target population as assessed by the ovitrap index and the mean number of eggs per trap. It is worth noting that during the last three weeks of the intervention, there were no eggs in the ovitraps of the treatment area.

## 7. Insect Pest Control Laboratory

Last but not least, the review article by Vreysen and colleagues (2021b) [35] on “The FAO/IAEA Insect Pest Control Laboratory: ten years (2010–2020) of research and development, achievements and challenges in support of the sterile insect technique” summarizes the catalytic progress which has been achieved on the development and refinement of the SIT of an important number of insect pests and disease vectors (fruit flies, lepidoptera, tsetse flies and mosquitoes) through the systematic and problem-solving R&D work carried out at the Insect Pest Control Laboratory. This R&D has significantly improved the efficiency and cost-effectiveness of the SIT towards the population management of insect pests of agricultural, veterinary and human health importance. In addition, it has resulted to the expansion of the SIT target species by including species such as *An. fraterculus*, *D. suzukii* and *Aedes* mosquitoes. The review also presents the collaborative research efforts carried out in the frame of Coordinated Research Projects (CRP), how the knowledge is transferred to FAO and IAEA Member States through Technical Cooperation Projects (TCP) as well as the major challenges faced.

## 8. Conclusions

The SIT has constantly proven its capacity to suppress, contain, prevent (re)introduction or even locally eradicate populations of selected key insect plant and livestock pests. However, there is also a continuous need to further refine and improve the efficiency as well as the cost-effectiveness. Several of the studies presented in the present Special Issue on “The sterile insect technique (SIT) and its applications” show that the (mass-)rearing, sex separation, irradiation, handling, packaging, and release process can be further refined including the quality control analysis of both the product and the process. In addition, it has been shown there is significant room for the expansion of the SIT to new target species of plant pests such as *D. suzukii* and even some potential hemipteran species.

During the last years, there has also been an increasing demand to develop and apply the SIT against mosquito vectors of human diseases such as *Ae. aegypti* and *Ae. albopictus*. Major advances have been made with a wide range of components of the SIT package, and this is reflected in the fact that several small-scale field trials are being carried out in several parts of the world against these two mosquito species with very encouraging results.

The major challenges for the further enhancement and deployment of SIT is the automation of as many steps as possible, the (faster) development of stable GSSs and the careful design and implementation of small-scale field trials using a phased conditional approach prior to any large-scale deployment. In all cases, the most important factor is the strong political will and commitment during all phases of an AW-IPM project with an SIT component.

## Data Availability

Not applicable.
